# Urine testing is associated with inappropriate antibiotic use and increased length of stay in emergency department patients

**DOI:** 10.1016/j.heliyon.2022.e11049

**Published:** 2022-10-12

**Authors:** Richard Childers, Ben Liotta, Jesse Brennan, Phoebe Wang, Jacob Kattoula, Thien Tran, Henry Montilla-Guedez, Edward M. Castillo, Gary Vilke

**Affiliations:** Department of Emergency Medicine, University of California, San Diego, San Diego, CA, United States

**Keywords:** Urinary tract infections, Asymptomatic bacteriuria, Antibiotic stewardship, Length of stay

## Abstract

**Background:**

Exposing patients with a low probability of disease to diagnostic testing with poor test characteristics leads to false positive results. Providers often act on these false results, which can cause unnecessary evaluation and treatment. The treatment of asymptomatic bacteriuria is discouraged, but it still frequently occurs in the inpatient setting; it is less studied in the Emergency Department (ED). In this study, we examine associations between urine testing, inappropriate antibiotic use, and length of stay in discharged ED patients at risk of urinary tract infection (UTI) misdiagnosis.

**Methods:**

A cohort of discharged ED patients at risk of UTI misdiagnosis was created by pulling visit information for patients presenting with abdominal pain, chest pain, headache, vaginal bleeding in pregnancy, and elderly females with weakness or confusion. Predictors of urine testing, and urinary tract infection treatment were determined with logistic regression analysis. A chart review of a representative sample of this cohort was then completed screening for the presence of urinary tract symptoms and urine culture results. Linear regression analysis was then used to generate an adjusted mean difference in length of stay between patients who had urine testing compared to those who did not.

**Results:**

About a quarter of chest pain and headache patients had urine testing, while approximately 75% of abdominal pain patients, vaginal bleeding in pregnancy, and elderly females with weakness or confusion did. Except for chest pain patients, the UTI treatment rate was more than double the positive culture rate, indicating overtreatment. A diagnosis of UTI is based on a combination of UTI symptoms and positive urine cultures, yet only about 15% of patients treated for UTI met these criteria. Lastly, in all chief complaint groups, the length of stay was significantly longer—30 min or more—for those who had urine testing compared to matched controls.

**Conclusions:**

In this observational study of patients at risk of UTI misdiagnosis, urine testing was associated with inappropriate antibiotic use and delayed discharge. There is pressure on providers to perform diagnostic testing, but in patients without specific UTI symptoms, urine testing might cause more harm than benefit.

## 1 Introduction

Clinicians often think that while more diagnostic information may not always be helpful, it will not hurt. However, harm from diagnostic information occurs when patients with a low pre-test probability of disease are exposed to testing. In these patients, there is likely to be a high rate of false positives. Though not indicative of disease, clinicians often act on the “positive” tests. Examples include false-positive d-dimers leading to unnecessary imaging and anti-coagulation, or false-positive cardiac stress-tests leading to unnecessary cardiac catheterization and percutaneous coronary intervention [1, 2]. Harms take two forms: the direct adverse effects of treatment (bleeding or procedural complications) and the opportunity costs (money and time) incurred.

The known benefit of antibiotics for urinary tract infection (UTI) are limited to patients with UTI symptoms; studies in this area enroll patients with some combination of dysuria, urinary urgency or frequency, suprapubic pain or flank pain [3, 4]. Antibiotic treatment for asymptomatic bacteriuria (ASB) has generally shown no benefit, and is recommended only for pregnant patients [5, 6]. Thus, in patients without urinary tract symptoms, testing should be uncommon, as the treatment with antibiotics for UTI in the absence of urinary tract symptoms has not been studied.

Despite the limited role for UT in patients without urinary symptoms, it is commonly ordered and can lead to adverse effects. Treatment of ASB in a systematic review of primarily inpatient studies was 45% [7]. A multi-center retrospective cohort study of 2733 hospitalized patients with ASB found an association between antibiotic treatment and longer hospitalization [8].

The drawbacks of inappropriate UT in the ED specifically are less studied. In a single-center study of 221 ED patients Khawcharoenporn reported an ASB treatment rate of 20%; the authors concluded that abnormal urinalysis results led to treatment of otherwise asymptomatic patients [9]. Shallcross reported in a UK cohort study that the majority of patients admitted to the hospital with an ED diagnosis of UTI did not have clinical or microbiological evidence of urinary tract disease [10]. There are data showing an association between testing, in general, and an increased ED length of stay (LOS) [11, 12]. Regarding urine testing specifically, a chart review of 287 discharged ED patients (all-comers over 12 consecutive days), using regression analysis, found that having a UA ordered was associated with an increased LOS though the actual difference was not reported [13].

We hypothesize that UT in patients without urinary symptoms causes harm in the form of unnecessary antibiotics and prolonged LOS. To test this hypothesis, we looked at the prevalence of urinary tract symptoms and positive urine culture results in ED patients who presented with symptoms unlikely to be due to UTI (chest pain, headache, etc) but who were treated for UTI at discharge. We also investigated the association of UT with ED LOS.

## 2 Methods

### 2.1 Study design

To examine the drawbacks to inappropriate UT, a historical cohort study and a chart review were completed at two academic EDs: an urban safety-net hospital and a tertiary academic medical center with a combined census of approximately 78,000 visits a year. The cohorts included patients presenting with chief complaints that were unlikely to be due to urinary pathology; thus, these patients, if exposed to UT, were at risk of misdiagnosis of UTI. The cohorts included patients presenting with any of the following chief complaints: abdominal pain, chest pain, headache, vaginal bleeding in pregnancy, or weakness or confusion or altered mental status in females greater than 65 years old. All adult discharged ED patients presenting with the above chief complaints between January 1, 2015, and December 31^st^, 2019, were identified from our electronic medical record.

Patients meeting three criteria were defined as having had UTI treatment. First, they had to receive a prescription for one of the following antibiotics frequently used to treat UTI: sulfamethoxazole-TMP, nitrofurantoin, fosfomycin, ciprofloxacin, cephalexin, or cefdinir. Second, patients receiving a second antibiotic were excluded. For example, patients receiving ciprofloxacin and metronidazole were likely being treated for diverticulitis, not UTI. Third, a urine culture must have been completed. In our system, urine cultures are automatically triggered by a positive urinalysis. Our hypothesis is that providers are diagnosing patients based on positive UT; thus, the completion of a urine culture implies positive UT. A positive urine culture was defined as >100,000 CFU/mL of a single uropathogen or >10,000 CFU/mL if the pathogen was group B streptococcus in pregnant patients [9].

### 2.2 Inappropriate antibiotic use

To investigate the appropriate use of antibiotics for UTI in these patients with non-UTI chief complaints, we performed a chart review of patients treated for UTI, recording the presence of UTI symptoms and results of urine culture. In trials of antibiotics for UTI, patients generally have UTI symptoms and positive testing [14, 15]. Of all types of UT, a urine culture is generally considered the strongest predictor of a UTI. Thus, patients diagnosed with UTI, but without UTI symptoms or positive urine cultures, are likely misdiagnosed. The term overtreatment, as used in this manuscript, indicates an unnecessary treatment unlikely to benefit the patient.

A representative sample of the cohort was reviewed. Sample size was set at a confidence level (power) of 95% with a confidence interval of 10 [16]. Charts were randomly selected from the entire cohort. In patients who received a UTI antibiotic, the ED provider note was reviewed for any of the following signs and symptoms: dysuria, urgency, frequency, suprapubic pain, flank pain, or CVA tenderness. In questionable cases, we would err on the side of coding cases as positive; this inclusive view on the presence of UTI symptoms biased the results against our hypothesis. To determine if our methods for identifying patients treated for UTI were accurate, we also noted if the indication for the antibiotic being given was, indeed, for UTI.

Abstractors included two undergraduate pre-medical students, two emergency medicine residents, an emergency medicine pharmacist, and an emergency medicine attending. When ambiguous chart elements were encountered by abstractors, the first and second authors conferred to make a final coding decision. The first and second author independently reviewed 25% of charts to assess interrater agreement. Abstractors were all study authors and were not blinded to the study hypothesis.

### 2.3 Length of stay differences

To examine the association between UT and LOS, a historical cohort study was completed. We compared the unadjusted mean LOS in those patients who received UT compared to those who did not. Linear regression analysis was used to provide an adjusted mean difference. Adjustment variables included age, race, gender, presence of fever (≥100.5), and presence of CT imaging when appropriate. Fever and CT imaging were used as markers of patient acuity. In the altered elderly female cohort, patients with fever were excluded; guidelines allow for treatment in this group of patients if there are signs of systemic infection such as fever [17].

In addition to the groups described above, we also included a cohort of patients who received either a complete blood count or a chemistry. Often, patients get “screening labs” which include either of these two blood tests. Some providers include UT as part of this. We were curious if adding a urine test affected the LOS in this group.

This study was reviewed and approved by the University of California San Diego Human Research Protections Program. This minimal risk study was granted a waiver of informed consent.

### 2.4 Data analysis

Descriptive characteristics were used to describe the cohort characteristics. They were also used to describe the prevalence of UTI symptoms and urine culture results among the sample selected who had been given a prescription for a UTI antibiotic. Univariable and multivariable logistic regression was used to examine predictors of receiving urine testing and UTI treatment. For the categorical variable race, p-values were not corrected for multiple comparisons. Linear regression was used to generate an adjusted mean difference in LOS between patients with and without UT. Confounders (age, gender, race, presence of fever, and presence of CT imaging) were pre-specified and forced into the model. Analyses were performed using SPSS for Mac, version 26 (IBM Corp., Armonk, NY).

## 3 Results

54,005 charts were screened for review. Cohort characteristics are described in Table 1. Of all the cohorts, patients presenting with chest pain had the lowest rate of UT (19.5%), but the highest rate of positive urine culture (14.2%). Almost three-quarters of abdominal pain, vaginal bleeding in pregnancy, and elderly females with confusion had UT. The rate of UTI treatment varied, but, except for chest pain patients, was at least double the rate of positive urine cultures indicating overtreatment. The rate of positive urine cultures in the pregnant cohort was relatively low at 1.3%; rates of asymptomatic bacteriuria in pregnant patients ranges from 1.9 to 9.5% [17]. It was also low for confused elderly females at 7.7%; generally, it ranges from 30 to 50% [18].

**Table 1:** Descriptive characteristics of discharged emergency department patients presenting with one of five chief complaints. SD = standard deviation, UCX = urine culture, UTI = urinary tract infection, LOS = length of stay.

**Chief Complaint Group (n)**	**Abdominal Pain (26,798)**	**Chest Pain (16,122)**	**Headache (7,763)**	**Vaginal Bleeding in pregnancy (2,161)**	**Elderly females with weakness or confusion (1,161)**
Mean Age (SD)	44.3 (17.0)	50.5 (16.9)	44.1 (16.4)	30.6 (6.1)	78.0 (8.9)
Female (%)	60.7	50.2	65.3	100	100
Race (%)					

White	48.4	51.0	47.3	39.1	61.0
Black	11.4	15.0	12.6	8.7	9.6
Asian	6.9	7.2	6.7	9.3	8.3
Pacific Islander	0.4	0.5	0.2	0.3	0.9
American Indian	0.2	0.2	0.3	0.3	0.2
Other	32.7	26.1	32.8	42.2	20.0
Underwent Urine Testing (%)	75.6	19.5	27.3	73.1	78.5
Positive UCX (%)	2.4	14.2	1.2	1.3	7.7
UTI antibiotic (%)	6.2	1.3	2.2	6.0	15.4
Median LOS (hours)	6.2	5.8	5.3	5.4	6.3

Table 2 illustrates the results of a logistic regression analysis examining predictors of urine testing and UTI treatment. Age was weakly predictive for both outcomes. Females were much more likely to get UT (OR 2.4; p < .001) and UTI treatment (OR 3.5; p < .001). Race was weakly predictive of UT and UTI treatment, but, compared to whites, blacks were less likely to get either testing (OR 0.88; p < .001) or treatment (0.74; p < 001) even when controlling for age and gender.

**Table 2:** Predictors of receiving urine testing and receiving an antibiotic for urinary tract infection using logistic regression analysis. OR = odds ratio.

**Predictor**	**Univariable OR (95% CI)**	**P-value**	**Multivariable OR (95% CI)**	**P-Value**
*Urine Testing*				
Age (Per Year)	1.004 (1.003–1.004)	<.001	1.0 (1.0–1.0)	<.001
Race				
Asian	1.2 (1.2–1.3)	<.001	1.1 (1.1–1.2)	<.001
Other	1.2 (1.2–1.2)	<.001	1.2 (1.2–1.2)	<.001
Pacific Islander	1.0 (.89–1.2)	.78	.92 (.81–1.1)	.26
American Indian	.93 (.78–1.1)	.39	.87 (.72–1.03)	.11
Black	.88 (.86–.90)	<.001	.90 (.87–.92)	.001
White	Reference	-	Reference	-
Gender				
Female (vs Male)	2.4 (2.4–2.4)	<.001	2.4 (2.3–2.4)	<.001
*UTI Treatment*				
Age (Per Year)	1.1 (1.0–1.0)	<.001	1.0 (1.0–1.0)	<.001
Race				
Asian	1.3 (1.2–1.4)	<.001	1.2 (1.1–1.3)	<.001
Other	1.2 (1.2–1.3)	<.001	1.3 (1.2–1.4)	<.001
Pacific Islander	1.0 (.74–1.5)	.81	.95 (.68–1.3)	.78
American Indian	.97 (.62–1.5)	.91	.92 (.59–1.4)	.71
Black	.74 (.69–.80)	<.001	.78 (.73–.85)	<.001
White	Reference	-	Reference	-
Gender				
Female (vs Male)	3.5 (3.3–3.6)	<.001	3.4 (3.3–3.6)	<.001

A chart review of a cohort sample show that most patients treated for UTI in our cohort are unlikely to benefit from this treatment (Table 3). Patients enrolled in trials for UTI treatment generally have urinary tract symptoms and positive culture results; yet only around 15% of the cohort treated for UTI met these criteria. The pregnant group was especially unlikely to benefit; none had symptoms and positive cultures, and only 1.8% had asymptomatic bacteriuria. However, the methods for coding cases for UTI treatment were not perfect; 8.8% of the abdominal pain cases our methods coded as being treated for UTI were given an antibiotic for a different disease. The most common reason for an erroneous coding of UTI antibiotic was ciprofloxacin given for enteritis. The error rate was lower for the other groups.

**Table 3:** Prevalence of lower urinary tract symptoms and urine culture results in ED patients, at risk of UTI misdiagnosis, who get a UTI antibiotic. This is a chart review of a sample of the overall cohort. The second column are the proportion of patients with both UTI symptoms and culture results and are the population who might benefit from antibiotic treatment. The last column indicates incorrect coding of cases, by our methods, for UTI treatment; the higher the number, the more error is introduced into our results. UTI = urinary tract infection, UCX = urine culture.

**Chief Complaint Group (N)**	**+ UTI symptoms + UCX (%)**	**+ UTI symptoms - UCX (%)**	**- UTI symptoms + UCX (%)**	**-UTI Symptom -UCX (%)**	**Incorrectly coded as UTI treatment (%)**
Abdominal pain (91)	13.2	44.0	8.8	34.1	8.8
Chest pain (67)	13.4	20.9	22.4	43.3	4.5
Headache (62)	16.1	24.2	24.2	35.5	3.2
Vaginal bleeding in pregnancy (55)	0	14.5	1.8	83.6	3.6
Elderly females with weakness or confusion (77)	13.0	20.8	19.5	46.8	0

The first and second author reviewed 88 of the cases to rate interrater agreement. Ten (11.4%) results were changed. In seven cases, the code was changed to positive UTI symptoms; in three cases the code was changed to negative UTI symptoms.

In all cohorts, UT was associated with a statistically significant longer LOS (Table 4). Groups with UT generally had a LOS 30–40 min longer than patients not exposed to UT. This difference was about 10–15% of the overall LOS. In the group of all patients who had blood testing, the difference was especially long at 70 min.

**Table 4:** The association of urine testing with length of stay in discharged ED patients at risk of UTI misdiagnosis. The adjusted mean difference was derived using multivariable linear regression analysis. Age, gender, race, presence of fever, and presence of abdominal CT imaging were controlled for.

**Chief Complaint Group (N)**	**Unadjusted Mean LOS (minutes) Urine Test Completed (95% CI)**	**Unadjusted Mean LOS (minutes) Urine Test Not Completed (95% CI)**	**Adjusted Mean Difference (minutes) (95% CI)**	**P-Value**
Abdominal pain (21,701)	346.1 (344.5–347.7)	298.8 (295.8–301.8)	32.4 (29.2–35.7)	<.001
Chest pain (12,688)	346.4 (342.1–350.7)	301.8 (299.7–303.9)	37.8 (32.8–42.8)	<.001
Headache (6,662)	337.1 (332.3–341.8)	284.8 (281.7–287.8)	48.2 (42.1–54.4)	<.001
Vaginal bleeding in pregnancy (1,994)	326.0 (320.9–331.1)	294.7 (285.6–303.9)	29.9 (19.9–39.9)	<.001
Elderly females with weakness or confusion (8,96)	345.4 (338.2–352.5)	308.8 (293.7–324.0)	35.9 (20.2–51.5)	<.001
Received CBC and/or blood chemistry (n = 169,306)	324.7 (323.6–325.8)	242.6 (241.9–243.2)	69.8 (68.5–71.2)	<.001

## 4 Discussion

This retrospective study found that many ED patients presenting with non-UTI complaints who are treated for UTI are likely to have been misdiagnosed. Further, urine testing in these patients was associated with a clinically significant increase in LOS. Over treatment of ASB in hospitalized patients is well-studied; however, this study adds to the scant information regarding discharged ED patients. Testing, in general, is associated with an increase in LOS for ED patients; this work isolates the association between UT specifically and an increase in LOS.

The prevalence of positive urine cultures in our population differed slightly from epidemiologic data. This is most likely due to differences in our population and methodology. Interestingly, patient presenting with chest pain had a higher rate of positive cultures compared to the other groups. This group had a lower rate of testing so we hypothesized that providers may have only ordered UT in chest pain patients if they had urinary symptoms. This could lead to a higher positive rate. Of course, it could be chance.

Guidelines are clear: only pregnant patients benefit from treatment of ASB [6]. However, one issue in the ED is the definition of asymptomatic. Most of the studies on the topic involve truly asymptomatic patients [5]. Patients enrolled at geriatric centers and outpatient clinics because they are asymptomatic are different than ED patients. ED patients are, almost by definition, symptomatic. They may not have urinary symptoms, but patients generally come to the ED for some sort of symptom. It is difficult to definitively disprove a causal link between vague symptoms and the possibility of a UTI.

One classic example of this scenario is confusion in older patients. Key texts recommend in older patients with confusion that a “urinalysis should be performed whether or not fever is present.” [19] This is contraindicated by guidelines which recommend treating ASB in cognitively impaired patients only if there are systemic signs of infection such as fever or hemodynamic instability [17]. While it is hard to disprove a causal relationship between positive UT and confusion in a demented patient, the benefit of antibiotic treatment in these populations can be studied. Randomized trials on this topic, if they show no benefit, might help change provider behavior and decrease unnecessary urine testing.

Providers may think that having a low threshold for UT is good for patients; however, in a patient without urinary symptoms, our work suggests getting UT is more likely to result in overtreatment and an increased LOS. Fear of malpractice, intolerance of uncertainty, pressure from patients and colleagues, and financial incentives: these are just some of the reasons providers overtest [20]. While addressing overuse is a Sisyphean task, this work provides a counterweight, however small, to the pressures encouraging providers to over test.

## 5 Limitations

This study was done at two different hospitals with different patient populations—a suburban tertiary referral center and an urban trauma center. However, these hospitals are within the same system and these results may not be generalizable to other emergency departments. Nevertheless, we suspect similar practices are common. In the systematic review cited in the introduction, 30 studies of ASB were identified and all of them found high rates of inappropriate antibiotic use [7]. While acknowledging publication bias, we are not aware of any studies that have not found significant over-treatment of ASB. Most studies are of hospitalized patients, but it would seem unlikely for the ED to be different.

Like any observational study, this work does not prove that UT in patients presenting with non-UTI related chief complaints causes inappropriate antibiotic use or increased LOS. UT may be associated with the type of provider who is generally slower or be associated with sicker patients who require more testing, and thus not be the causative factor in prolonging LOS. However, these associations were found in all pre-specified groups, the magnitude of the associations were large, and these findings have face-validity. It makes sense; it often takes time for patients to provide a urine sample. Regarding causation of antibiotic prescriptions, other authors have also concluded that false-positive UT, in the absence of symptoms, leads to inappropriate treatment of ASB [9]. This hypothesis also has face-validity: it is difficult for ED providers to ignore a positive urine test once it is available and documented in the medical record, so it follows that positive testing drives antibiotic prescription in asymptomatic patients.

We studied cohorts, based on chief complaint, at risk of UTI overdiagnosis. Patients presenting with non-UTI chief complaints like headache can certainly have an underlying diagnosis like pyelonephritis. We do not propose providers never get UT in patients presenting with a non-UTI chief complaint; regardless of the chief complaint, providers should order urine testing when it is felt it might change management. This was just a method to estimate the prevalence of inappropriate testing.

Emergency providers do not have urine culture results at the time of diagnosis and rely on urine dipstick testing and microscopy. The definition of a “positive” dipstick and urinalysis can get complicated; nitrate, leukocyte esterase, pyuria, hematuria, and gram stain all have their own test characteristics. However, these test characteristics are generally judged by the gold standard of urine culture [21, 22]. Thus, for simplicity, we used urine culture as a surrogate to determine positive urine testing. It is likely some of the patients we labeled as being inappropriately treated with antibiotics had positive UT at the visit, but the culture was later negative.

Our methods assume that patients who present with non-UTI chief complaints will not have specific urinary tract symptoms; however, 30–50% of patients treated for UTI in our cohort were found to have urinary tract symptoms in chart review. However, it should be noted this is likely an overestimate. The methods were purposely inclusive to bias against our hypothesis. Evidence for our inclusivity is that the majority of those coded as having UTI symptoms had negative urine cultures (Table 3). In any event, the group that coded positive for both symptoms and urine culture was smaller at about 15%. This represents significant overtreatment.

## 6 Conclusion

In patients presenting to the ED with non-UTI related chief complaints, UT is associated with inappropriate antibiotic use and prolonged LOS. Only ∼15% had localizing symptoms and positive cultures. Compared to matched controls, patients who had UT spent around 30 min longer in the ED. The known benefit of antibiotics for UTI occurs in patients with a combination of urinary tract symptoms and positive test results. Until research shows a benefit to treating ED patients with positive UT but no UTI symptoms, testing in these patients should be avoided.

## Declarations

### Author contribution statement

Richard Childers: Conceived and designed the experiments; Performed the experiments; Analyzed and interpreted the data; Wrote the paper.

Ben Liotta: Conceived and designed the experiments; Performed the experiments, Wrote the paper.

Jesse Brennan and Edward M. Castillo: Analyzed and interpreted the data.

Phoebe Wang: Performed the experiments; Wrote the paper.

Jacob Kattoula, Thien Tran and Henry Montilla-Guedez: Performed the experiments.

Gary M. Vilke: Conceived and designed the experiments; Wrote the paper.

### Funding statement

This research did not receive any specific grant from funding agencies in the public, commercial, or not-for-profit sectors.

### Data availability statement

Data will be made available on request.

### Declaration of interest’s statement

The authors declare the following conflict of interests: Dr. Vilke is a paid legal consultant.

### Additional information

No additional information is available for this paper.
